# A Case of Hour-Glass Contraction of the Womb, with Retention of Placenta and Followed by Internal Hemorrhage

**Published:** 1886-06-20

**Authors:** J. McF. Gaston

**Affiliations:** Professor of the Principles and Practice of Surgery in the Southern Medical College, Atlanta, Georgia


### Southern Medical Record.

A MONTHLY JOURNAL OF PRACTICAL MEDICINE.

☞All Communications must be addressed to R. C. Word, M. D., Managing Editor.

"*Quicquid Præcipies Esto Brevis.*"

Vol. XVI. ATLANTA, GA., JUNE 20, 1886. No. 6.

### ORIGINAL AND SELECTED ARTICLES.

### A CASE OF HOUR-GLASS CONTRACTION OF THE WOMB, WITH RETENTION OF PLACENTA AND FOLLOWED BY INTERNAL HEMORRHAGE.

By J. McF. Gaston, M. D.,

Professor of the Principles and Practice of Surgery in the Southern Medical College, Atlanta, Georgia.

Mrs. A. B. C——, about twenty-one years old, and married less than a year, was taken in labor with her first child on the morning of the 27th of May, 1886, and soon afterwards the amniotic fluid commenced flowing away, little by little. I was called to her at 1 o'clock p. m., and, upon examination, ascertained that there was a species of tonic contraction which pressed the uterine contents downwards, but without sufficient dilation of the os uteri to admit of the introduction of the point of the index finger. From the general outline of the prominence felt through the vaginal walls I was able to distinguish a vertex presentation, and hence felt safe in leaving her with instructions to have me notified so soon as periodic pains should be developed. Between 5 and 6 o'clock, being recalled, I learned that the water had continued to dribble away, and that the pains were recurring at intervals, while the bowels had been evacuated twice during the day, but having no assurance that the bladder had been emptied, the patient was advised to make an effort to effect this, which secured the desired result. The dilatation of the mouth of the womb was found to be progressing favorably, and with the vivid impression left upon my mind by the favorable result in Dr. Hiram Corson's three thousand and thirty-six cases of labor, I concluded to let Nature take her course, unless something should demand my interference. Notwithstanding the repeated calls of the patient for relief from her sufferings as the labor progressed, I desisted from the use of chloroform, which has been often resorted to in my obstetric practice with primiparas. The resistance of the soft parts was such that the vertex was exposed at the vulva and the whole craneal tumor seemed to protrude in advance of the coccyx, with the distended perineum surrounding it at each recurring pain, for nearly an hour before its final expulsion. The vaginal secretion being abundant, and the head becoming elongated by the graduated compression of my hands around the protuberance, the delivery took place without any laceration at a quarter before 9 o'clock p. m.

Having ligated the umbilical cord at two points, and divided it between with scissors, as is my custom, notwithstanding the latter-day practice of some to dispense with a ligature, the robust child was delivered to the nurse for her attentions, when I proceeded to care for the mother. Upon palpation over the hypogastric region the womb was found to be contracted, and a reasonable time having elapsed without the expulsion of the placenta from its cavity, as was ascertained by a vaginal exploration, I resorted to the gentle grasping of the womb with my hand to promote further contraction, and awaited the result until a half hour had elapsed from the delivery of the child. Perceiving that no progress had been made towards the expulsion of the placenta, I then grasped the womb with my left hand, while with the fingers of the right I sought to bring down one margin of the placenta still entirely within the uterine cavity. In this I succeeded, but found there was such resistance to the descent of the mass after a portion had been brought down into the vagina that my fingers were carried upward along its surface, when it was discovered that a circular constriction of the uterine fibers held a segment of the placenta so as to resist traction upon the larger part below it. Using the points of my fingers for dilatation, and employing now the left hand for moderate traction, the placenta was removed with its membranes; but, upon further examination, it was very evident that the contraction had not yielded entirely to this gentle dilatation, though it had allowed the retained portion of placenta to be removed. Ascertaining that the body of the womb presented a general outline upon external palpation which seemed satisfactory, the broad abdominal bandage was applied, and a cup of tea ordered for the patient, which she took with a relish.

Leaving the house at half-past 9 o'clock, with the injunction to notify me if anything unusual occurred, I was summoned to return at 11 o'clock with the statement that the parturient had become sick and vomited, with great sense of weakness. My first move, upon approaching her bedside, was to pass the hand over the uterine region, and this organ was discovered to be enlarged to about the size at third or fourth month of pregnancy, and passing the fingers of my right hand within the vagina it was found full of blood clots, while the site of the former abnormal contraction seemed to have shut a considerable amount of blood within the uterine cavity, above it. Brandy was immediately ordered to be given freely. While I proceeded to dilate the contractin and remove the clots with my right hand, the left was used in grasping the body of the womb from without, so as to secure its general and uniform contraction, thus averting what threatened to be a serious result.

In other cases of hour-glass contraction which I have encountered, it has been noted that there exists a relaxation of the uterine tissues above the constricting band ; and those who have read my reports of those cases in the New Orleans Medical Journal, some years ago, will recall that I impressed upon the accouchier the great importance of complete dilatation of the contraction. The omission to follow this course in the present occasion was undoubtedly the cause of the internal hemorrhage that occurred, and which demanded prompt measures for its relief, requiring ergot to complete the work

